# Mesenchymal Stem/Stromal Cells seeded on cartilaginous endplates promote Intervertebral Disc Regeneration through Extracellular Matrix Remodeling

**DOI:** 10.1038/srep33836

**Published:** 2016-09-22

**Authors:** Catarina Leite Pereira, Graciosa Q. Teixeira, Cláudia Ribeiro-Machado, Joana Caldeira, Madalena Costa, Francisco Figueiredo, Rui Fernandes, Paulo Aguiar, Sibylle Grad, Mário A. Barbosa, Raquel M. Gonçalves

**Affiliations:** 1 ICBAS, Instituto de Ciências Biomédicas de Abel Salazar, Universidade do Porto, Porto, 4050-313, Portugal; 2 Instituto de Investigação e Inovação em Saúde, Universidade do Porto, Porto, 4200-135, Portugal; 3 INEB, Instituto de Engenharia Biomédica, Universidade do Porto, Porto, 4200-135, Portugal; 4 IPATIMUP–Institute of molecular pathology and immunology of the university of Porto, Porto 4200-135, Portugal; 5 UMIB–Unit for Multidisciplinary Biomedical Research of ICBAS, Universidade do Porto, Porto, 4050-313, Portugal; 6 IBMC–Instituto de Biologia Molecular e Celular, Porto 4150-180, Portugal; 7 AO Research Institute Davos, Davos, 7270, Switzerland

## Abstract

Intervertebral disc (IVD) degeneration is characterized by significant biochemical and histomorphological alterations, such as loss of extracellular matrix (ECM) integrity, by abnormal synthesis of ECM main components, resultant from altered anabolic/catabolic cell activities and cell death. Mesenchymal Stem/Stromal Cell (MSC) migration towards degenerated IVD may represent a viable strategy to promote tissue repair/regeneration. Here, human MSCs (hMSCs) were seeded on top of cartilaginous endplates (CEP) of nucleotomized IVDs of bovine origin and cultured *ex vivo* up to 3 weeks. hMSCs migrated from CEP towards the lesion area and significantly increased expression of collagen type II and aggrecan in IVD, namely in the nucleus pulposus. Concomitantly, hMSCs stimulated the production of growth factors, promoters of ECM synthesis, such as fibroblast growth factor 6 (FGF-6) and 7 (FGF-7), platelet-derived growth factor receptor (PDGF-R), granulocyte-macrophage colony-stimulating factor (GM-CSF) and insulin-like growth factor 1 receptor (IGF-1sR). Overall, our results demonstrate that CEP can be an alternative route to MSC-based therapies for IVD regeneration through ECM remodeling, thus opening new perspectives on endogenous repair capacity through MSC recruitment.

The unique and complex structure of the intervertebral disc (IVD) confers to it exclusive features, such as the capacity to support the whole body weight and a wide range of movements/loadings on the spine. The IVD is an avascular organ composed of a gel-like central part, the nucleus pulposus (NP), surrounded by a lamellar fibrous structure, the annulus fibrosus (AF), and the cartilaginous endplates (CEP), which link each disc to the adjacent vertebral bodies^1^. With ageing, IVD undergoes a degeneration process, in which mismatch between anabolic and catabolic processes orchestrate an alteration of matrix composition, which differs from extracellular matrix (ECM) of healthy IVD^2^. During IVD degeneration several histomorphological changes occurs, including NP fibrosis, loss of lamellar organization of the AF, and increased cell death and senescence. In addition, the ability of the IVD to support mechanical forces and to provide flexibility and mechanical stability to the spine becomes compromised due to a loss ECM integrity, caused by abnormal synthesis of its main components–collagen, proteoglycans–and a great loss of water content. Altogether, these alterations culminate in the loss of IVD biological function^3^.

Current treatments for IVD degeneration focus on painful degenerative discs and involve conservative approaches or in more severe situations, surgical procedures such as spine fusion or IVD replacement. However, these strategies can affect spine biomechanics and are not able to restore the IVD biological function; besides triggering degeneration of adjacent discs^4^. Alternative strategies based on biomechanically-competent hydrogels^5^ that share similar composition to disc ECM have been attempted, but failed mostly due to material extrusion from AF after implantation^6^. From another perspective, cell-based therapies for degenerated IVD have quickly grown over the past years, namely using Mesenchymal Stem/Stromal Cells (MSCs)^7^. MSCs were shown to differentiate into NP-like cells^8^,^9^. *In vitro*, MSC co-cultured with NP cells associate with both up-regulation of SOX-9 and ECM proteins, in particular aggrecan (Agg) and collagen type II (Col type II), dependent on direct cell-cell contact^8^,^10^,^11^. *In vivo*, MSCs were already transplanted to IVD in different animal models, as extensively reviewed by Sakai and Andersson^12^. The promising results obtained from those studies, such as the higher expression of Col type II and disc height recovery^12^, have encouraged clinical trials based on MSC transplantation as treatment for degenerated IVD and low back pain^13^,^14^. The published clinical trials have shown significant pain reduction, a partly recovery of disc hydration, and few signs of IVD regeneration^15^. In fact, cell transplantation through the AF faces several problems: first, the injection itself can trigger IVD degeneration^16^; second, the high IVD pressure can lead to cell leakage and trigger osteophyte formation^17^; and third, the hostile environment of degenerated IVD can induce cell death post-injection^18^.

Therefore, the CEP appears as an alternative route to deliver therapeutic agents to the degenerated IVD. By using a new surgical method, the transpedicular approach, Vadalá *et al*.^19^,^20^ showed that the NP can be approached through the CEP via pedicle without affecting the spinal canal and the neural foramina. This route can be an alternative path to reach the NP, without AF disruption, which is one of the major problems of IVD surgical approaches.

In the past few years, several arguments were raised stressing the role of the CEP in accessing the NP to treat IVD degeneration. Cells with stem cell-like characteristics were described in the CEP of degenerated human IVDs^21^. CEP stem cells were shown to differentiate into osteogenic and chondrogenic lineages, in a rabbit IVD degeneration model, suggesting their applicability in NP tissue engineering^22^,^23^. Moreover, Henriksson, *et al*.^24^,^25^ described for the first time the presence of slow cycling cells and a stem cell niche around adult IVD region and suggested that these cells can migrate towards the AF and inner parts of the IVD. On the other hand, Illien-Jünger and colleagues^26^ demonstrated that *ex vivo* cultures of IVDs in degenerative conditions secrete chemokines that specifically recruit MSCs and not fibroblasts. Furthermore, our own group showed that incorporation of the chemoattractant Stromal Cell Derived Factor-1 (SDF-1) into a hyaluronic acid hydrogel, promotes MSC migration from the CEP to the NP and AF^27^.

In this study, we investigated the role of human MSCs (hMSCs) seeded on CEP in IVD tissue remodeling, using long-term *ex vivo* cultures of nucleotomized IVDs. We hypothesized that repopulation of the IVD with healthy cells has the potential to restore tissue homeostasis and reverse the degenerative process. Although an enormous challenge, a strategy that could stop/revert IVD degeneration, without damaging the AF, would be of great relevance.

## Results

### IVD long-term organ culture: metabolic activity, cell proliferation and hMSC migration

**Figure 1:**
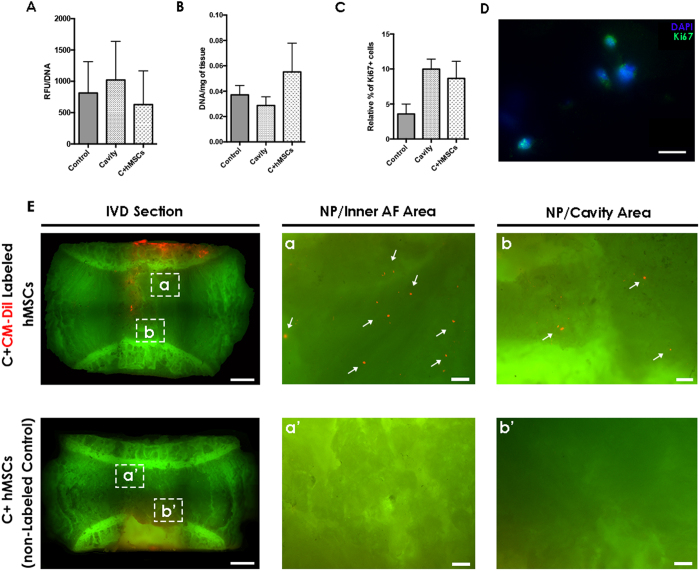
Metabolic activity, DNA, Cell proliferation and hMSCs migration in the IVD tissue after 21 days of culture. Control, cavity and C + MSCs were compared at day 21 for: **(A)** metabolic activity, no differences were observed between the groups; **(B)** DNA content, a slight increase in DNA was observed in the C + hMSCs group; **(C)** cell proliferation quantification: higher cell proliferation was observed in both lesioned groups (cavity and C + hMSCs). All depicted results are presented as M/D (n = 3-7) without significant differences (Mann-Whitney test). **(D)** representative image of proliferative Ki67+ cells in the IVD tissue is presented (scale bar: 10 μm). **(E)** representative image of CM-Dil-labeled hMSCs and the control with non-labeled hMSCs after 21 days in IVD organ culture (scale bar: 2000 μm / magnified image: scale bar: 200 μm). CM-Dil labeled hMSCs can be identified in red in the IVD tissue at day 21.

To investigate the effect of CEP-seeded hMSCs on the ECM remodeling of the IVD, whole organ cultures of nucleotomized discs from bovine origin were used as a model, similarly to our previous study^27^. hMSCs were seeded on the discs CEP and maintained in culture for 21 days. First, hMSC viability and survival in IVD culture media (supplemented with 2% Fetal Bovine Serum (FBS)) was confirmed by Annexin/Propidium Iodide staining (see supplementary data). After 21 days, the different conditions (control, cavity and C + hMSCs) were compared in terms of metabolic activity, DNA content and cell proliferation. Tissue/cells metabolic activity was evaluated by resazurin assay and showed a slight increase per cell in the cavity group (1021 ± 616 RFU/μg of DNA), although no significant differences were observed when compared to control (813 ± 500 RFU/μg of DNA) and C + hMSCs group (628 ± 538 RFU/μg of DNA) (Fig. 1A).

**Figure 2:**
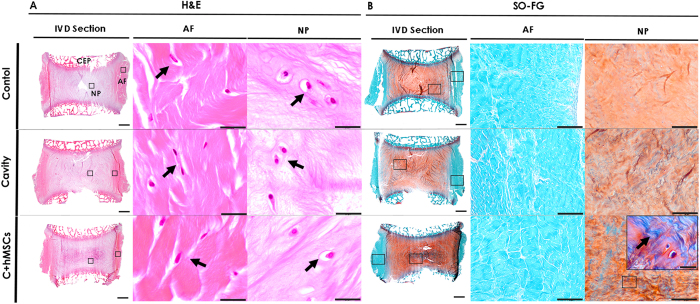
Histological analysis of the IVD tissue after 21 days of culture. Sagittal sections of the control, cavity and C + hMSCs were stained with **(A)** H&E and **(B)** SO-FG. One representative donor was depicted. In the IVD whole section (scale bar: 2000 μm), CEP, NP and AF regions of IVD can be distinguished. Magnifications of NP/AF cells are presented (scale bar: 20 μm) in the H&E staining as well as magnifications of the whole AF/NP tissue in SO-FG (scale bar: 200 μm). Collagens are visualized by green/blue (arrow) color while GAG are observed in orange color. A higher intensity of orange color in the C + hMSCs group NP suggested a higher proteoglycan content in the tissue of this experimental group.

The DNA content of nucleotomized (0.03 ± 0.01 μg/mg) and control (0.04 ± 0.01 μg/mg) discs was similar, but a slight increase in the C + hMSCs group was observed (0.06 ± 0.04 μg/mg) (Fig. 2B). Cell proliferation was additionally evaluated by Ki67 positive expression using immunofluorescence (IF). In control IVDs, 4 ± 2% Ki67^+^ cells were found, while a slight increase in cell proliferation was observed in both cavity (10 ± 2% Ki67^+^ cells) and C + hMSCs groups (9 ± 4% Ki67^+^ cells) (Fig. 1C,D). Although a tend to increase in cell proliferation was observed in both nucleotomized groups, the values were out of statistical differences. The presence of hMSCs in the IVD tissue at day 21 was confirmed in a mosaic image of the whole IVD obtained by a high content screening system (InCell Analyzer 2000), in which CM-Dil red-labeled hMSCs could be identified (Fig. 1E-a,E-b (white arrows)). A control IVD with non-labeled hMSCs was also imaged (Fig. 1E-a′b′). These results showed that the majority of hMSCs remained on the CEP (red fluorescence), and a small proportion migrated throughout the tissue (about 3%, estimated by quantification of a central sagittal section of the IVD).

Since hMSC migration is mostly related with metalloproteinases (MMPs) production, MMP2 and MMP9 present in the organ culture media were evaluated by gelatin zymography. This technique allows for the detection of both active and inactive forms of MMPs. The results showed a higher activity of MMP2, both pro- and active form, in the C + MSCs group, while MMP9 activity was absent (see Supplementary Data). Nevertheless, the cavity group (without hMSCs) also revealed higher amounts of MMP2, so it was not possible to conclude whether MMP2 was produced by hMSCs or IVD cells, stimulated by the presence of hMSCs.

### Histological analysis of the IVD after long-term culture

The impact of hMSCs seeded on CEP in nucleotomized IVD after 21 days of culture was first evaluated by histology using hematoxylin/eosin (H&E) and safranin-O fast-green (SO-FG) staining. Representative sagittal sections of decalcified discs are depicted in Fig. 2. H&E staining allowed the observation of different and interrelated areas of the IVD, the CEP on the top and bottom of the IVD, the IVD central part (NP) and the surrounding fibrous, AF (Fig. 2A). A higher cell density was observed in the AF when compared with the NP area. Typical cells of the AF (‘fibroblast-like’) and NP (‘chondrocyte-like’) could be distinguished, as highlighted by the black arrows. At day 21, it was difficult to observe the lesion area as a result of tissue swelling (Fig. 2A,B). Nonetheless, when comparing tissue morphology in different conditions, a more organized structure was observed in control discs, while in the lesioned groups (cavity and C + hMSCs), namely in the NP, ECM fibers appeared to be more wrinkled, ruptured and disorganized. SO-FG staining allowed the distinction between peripheral AF (in blue), rich in collagen, and the inner AF and NP, containing mostly proteoglycans (in orange) (Fig. 2B). AF structure was maintained in all groups. The tenuous orange staining observed in the NP area of the cavity group, suggested the loss of proteoglycans, possibly as a result of the injury process. On the other hand, a stronger orange staining in the C + hMSCs group suggests for higher proteoglycan deposition in the NP (Fig. 2B). Furthermore, some areas of intense bluish-green were identified in the NP, indicating the presence of collagens (Fig. 2B, magnified image, highlighted by the arrows). The presence of collagen staining in the NP (bluish-green areas) increased with hMSC treatment. Moreover, we excluded the presence of calcifications resulting from hypothetical hMSC differentiation into osteogenic lineage by alizarin staining (see Supplementary Data).

### Sulphated Glycosaminoglycans and Collagen type II quantification in the IVD tissue

**Figure 3:**
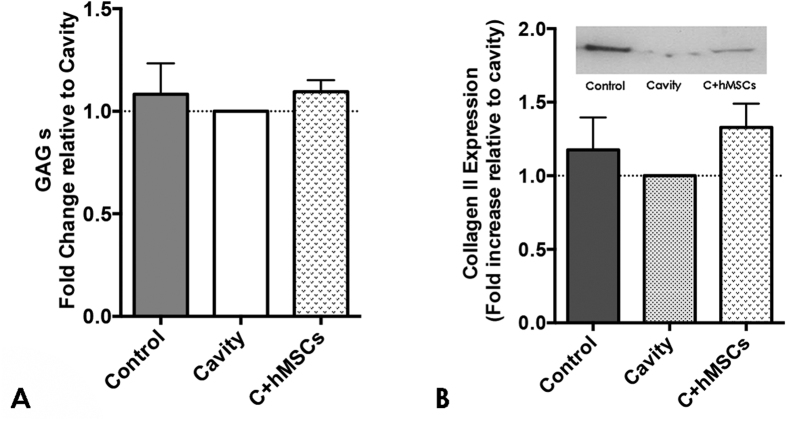
GAG and Collagen type II quantification in the NP tissue at day 21. The NP tissue of the three experimental groups was digested and afterwards analyzed for GAG and Col type II content. **(A)** Fold increase in GAGs content. GAG content was similar between the groups (n = 4). **(B)** Fold increase in Col type II and a representative image of a blot. Col type II content was determined by WB and values normalized to the total protein and presented as fold increase to the cavity group; a slight increase in Col type II was observed in the C + hMSCs group compared with the cavity (no statistically significance in raw data, Mann-Whitney test (n = 7)). Results are presented as M/D.

Sulphated Glycosaminoglycans (GAG) and Col type II were also analyzed in the NP of IVDs in the different groups, upon tissue digestion. GAG content was similar between all the groups (Fig. 3A) and no significant differences were observed. Col type II, quantified by western blot (WB), a slight increase in the C + hMSCs group was observed, but no significant statistical differences were detected compared with the control and cavity groups (Fig. 3B).

### Aggrecan expression in the IVD tissue

**Figure 4:**
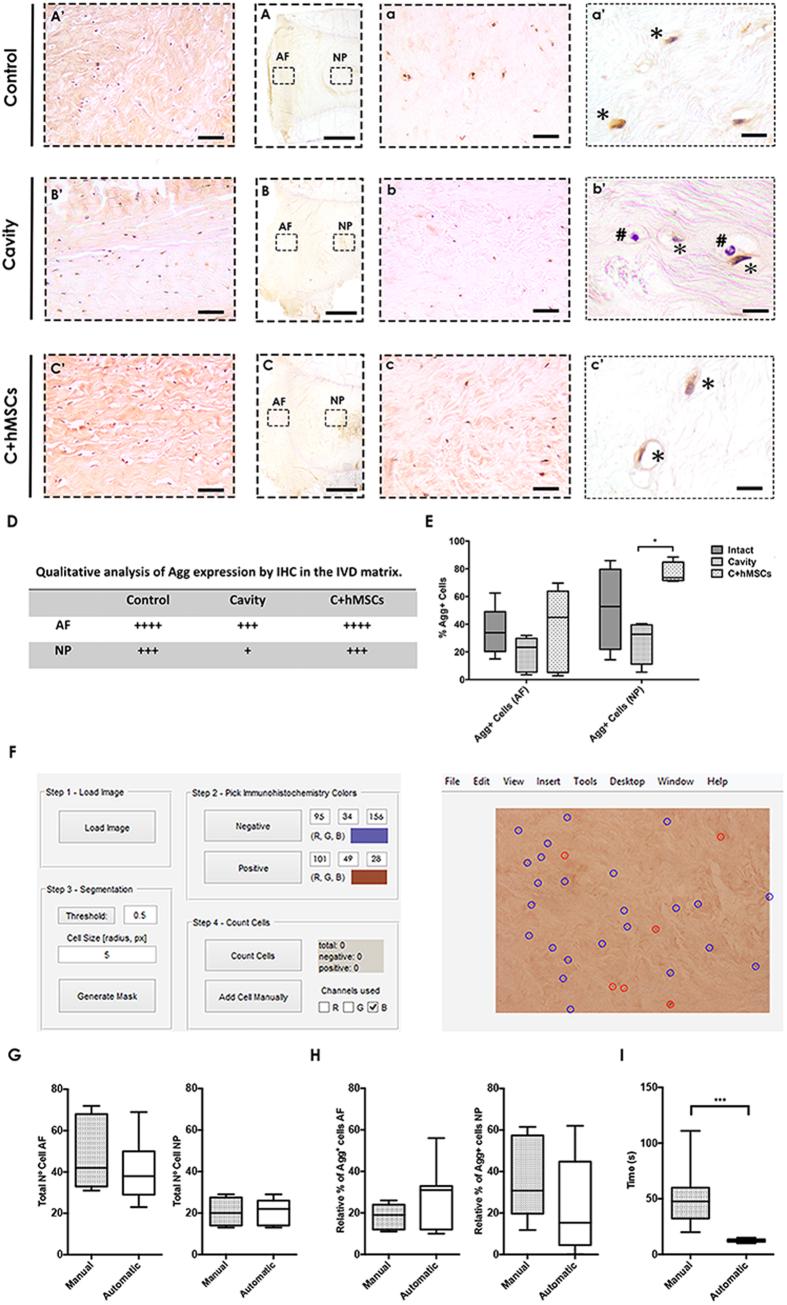
Aggrecan expression and quantification in the IVD tissue at day 21. Agg expression in the tissue was evaluated by IHC and displayed both matrix and cellular staining. **(A**–**C)** Overall Agg expression in the tissue. One representative donor is presented (scale bar-2000 μm). **(A′**–**C′)** Magnified representative images of Agg matrix and celular expression in the AF area (scale bar-200 μm). **(a,b)** Magnified representative images of Agg matrix and celular expression in the NP area (scale bar-200 μm). **(a′**–**c′)** High magnification images of cellular Agg expression in the NP (scale bar 20 μm). **(D)** Qualitative analysis of Agg matrix expression both in the AF and NP area. Cavity group displayed a less Agg matrix expression when compared to the control and the C + hMSCs group. **(E)** Quantification of Agg cellular expression (Agg+ cells were quantified using ImmunoCellCount software). Agg+ cells could be distinguished by a characteristic brown color, in co-localization with the hematoxylin purple staining. A significant reduction of Agg expression is observed in the cavity group in both AF and NP areas, while a significant increase is observed in the presence of hMSCs. Results are presented as box-and-whiskers plots (n = 5). **(F)** ImmunoCellCount software. Image on the left side, GUI where the settings can be established and on the right side, the image after the classification. Comparison between manual assessment of Agg deposition vs automatic using the ImmunoCellCount software was performed in both AF and NP, concerning: **(G)** Total number of cells; **(H)** Relative % of Agg+ cells; **(I)** Time of the analysis. No significant differences were observed between the two methods, but a there was a significant improvement in the time consumed for image analysis. Results are presented as box-and-whiskers plots (n = 5). Statistical analysis was performed using Mann-Whitney test (*p < 0.05; ***p < 0.001).

The expression and distribution of aggrecan (Agg) (the most abundant proteoglycan) in the IVD tissue was investigated *in situ* using immunohistochemistry (IHC) (Fig. 4A). Agg displayed both matrix and cellular staining after 21 days in culture in the IVD tissue (Fig. 4A–C). Concerning matrix staining, Agg expression was more intense in the AF (Fig. 4A′–C′) when compared with NP (Fig. 4a–c). In addition, a higher Agg expression was observed in both control and C + hMSCs groups in comparison to the cavity, particularly in NP where the differences were more accentuated (Fig. 4D). Regarding the Agg cellular staining, this was associated with a localized protein accumulation in the cell surroundings or even cell co-localization. Agg + cells adopted a brownish (*) color while negative cells were purplish (#), allowing distinction between the two classes (Fig. 4a′–c′). The total number of cells and the number of Agg+ cells were determined (Fig. 4E) using a custom-made software ImmunoCellCount (Fig. 4F) (see Supplementary Data). First the manual and automatic quantification of total number of cells were compared and no significant differences in the number and percentage of Agg+ cells, in both AF and NP was observed (Fig. 4G,H). Nevertheless, the time required for manual versus ImmunoCellCount-assisted calculation of Agg+ cells was longer, taking about 4.2 times more counting cells manually than with the program (Fig. 4I) (***p < 0.001). Still, the software presents some limitation in the identification of elongated cells, which can be easily overcome with the manual addition tool or by alteration of the cell size/threshold parameters. Moreover, false positive/negative counts can occur. Nevertheless, we estimate that the frequency these errors is around 5%, which encourages the use of the software to count Agg+ cells in IHC sections.

The frequency of Agg+ cells was slightly higher in the NP of control discs, comparing to the AF (52 ± 30% vs 35 ± 18%, respectively). A decrease of Agg+ cells was observed in cavity discs both in the AF (19 ± 13%) and NP (28 ± 16%). In the presence of hMSCs, the proportion of Agg+ cells increased in the AF (37 ± 30%) and was significantly higher (*p < 0.05) in the NP (77 ± 8%), when compared with the cavity group (Fig. 4E).

### Collagen type II expression in the IVD tissue

**Figure 5:**
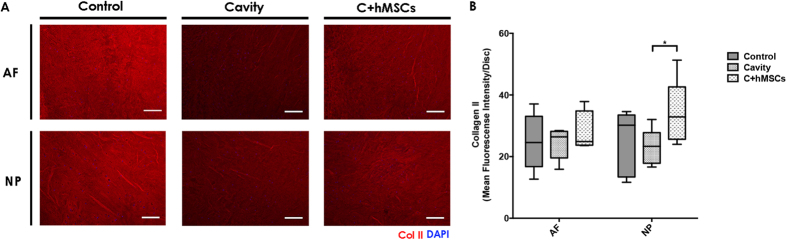
Collagen type II expression and quantification in the IVD tissue. At day 21 sagittal sections of control, cavity and C + MSCs) were analyzed for Col type II expression and distribution by IF. **(A)** Col type II expression in the tissue. One representative donor is presented (scale bar-200 μm). **(B)** Col type II expression was quantified using IntensityStatisticsMask Software (see supplementary data). A significant increase in Col type II expression was observed in the presence of hMSCs, in the NP area. Results are presented as box-and-whiskers plots (n = 5). Statistical analysis was performed using Mann-Whitney test (*p < 0.05).

Col type II expression and distribution *in situ* was also investigated in the IVD tissue using IF (Fig. 5A) and quantified by fluorescence intensity (see Supplementary Data). Col type II mean fluorescence intensity (MFI) in NP and AF was calculated separately. Col type II intensity in the AF was similar between all the groups tested: 25 ± 9 (control), 24 ± 5 (cavity) and 28 ± 7 (C + MSCs); while in NP, hMSCs significantly increased (*p < 0.05) the expression of Col type II (34 ± 11), when compared with cavity (23 ± 6) (Fig. 5B).

### Collagen type I expression in the tissue

**Figure 6:**
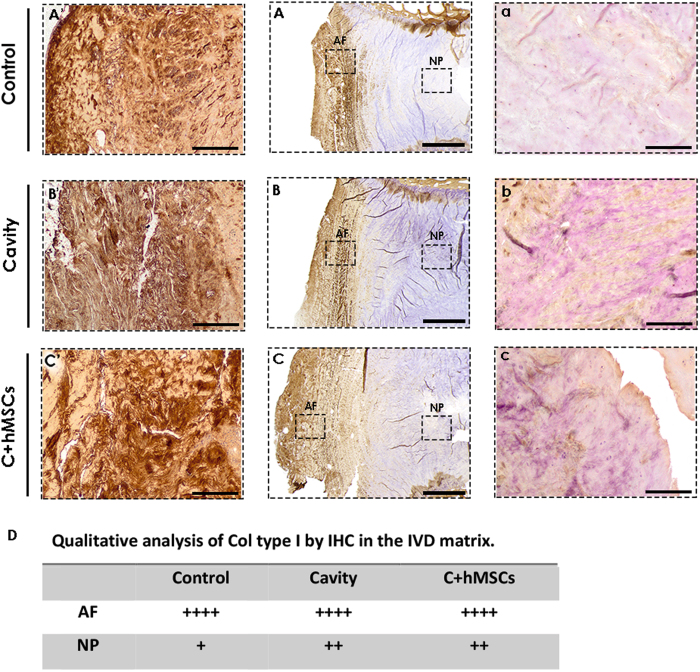
Collagen type I expression in the IVD tissue. At day 21 sagittal sections of control, cavity C + MSCs were analyzed for Col type I expression and distribution by IHC. **(A**–**C)** Overall expression of Col type I in the IVD tissue. One representative donor is presented (scale bar-2000 μm). Col I expression was more pronounced in the outer AF area, decreasing towards the NP central area (n = 3). **(A′**–**C′)** Magnified images of the AF area (scale bar-500 μm); **(a**–**c)** Magnified images of the NP area (scale bar-500 μm). **(D)** Qualitative assessment of col type I expression in the IVD matrix. A Higher expression was observed in lesion groups (cavity and C + hMSCs).

Col type I expression and distribution in the IVD tissue was also analyzed by IHC (Fig. 6A–C) and qualitatively analyzed (Fig. 6D). An intense expression of Col type I was found in the outer AF area decreasing gradually towards the NP central area. Overall the expression in AF areas was similar between the groups (Fig. 6A′–C′), while in the NP area, an increase of Col type I expression was noticed in both groups with lesion (cavity and C + hMSCs) when comparing to the control (Fig. 6a–c). Furthermore, no noticeable differences in Col type I expression were found in the NP of both cavity and C + hMSCs group.

### IVD tissue ultrastructure and collagen fiber quantification

**Figure 7:**
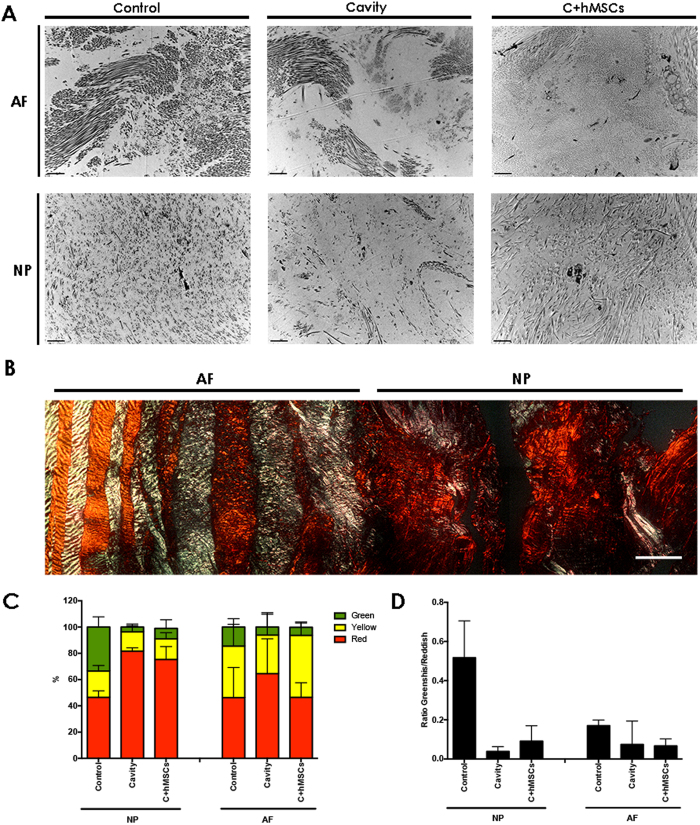
IVD ultrastructure and Collagen Fibers quantification. At day 21, controls and both cavity and C + hMSC ultrastructure were analyzed by TEM. **(A)** One representative donor is presented (scale bar-2 μm). Images were analyzed and scored for the different parameters (Table 1, (n = 3)). A loss of lamellar organization as well as ECM matrix was observed in the cavity group. Density levels seem to be recovered in the presence of hMSCs. In both conditions, cavity and C + hMSCs, a higher variability in the Coll fibers was observed, suggesting ECM remodeling. **(B)** Histological analysis of IVD stained using picrosirius red and visualised by polarised light microscopy (representative image of 1 disc). **(C)** Quantification of the relative percentage of collagen fibers (n = 3). **(D)** Ratio greenish/reddish (mature/immature) collagen fibres. The ratio of greenish/reddish fibers was very similar in the AF area while in the NP, where the lesion was previously performed, this ratio decreased in the cavity group and slightly increased in the presence of hMSCs.

The ultrastructure characterization of the IVD was observed by transmission electron microscopy (TEM) allowing a more profound visualization of matrix/cell alterations that occur during the degenerative process. Both AF and NP ultrastructure of the IVD of each group were imaged after 21 days in culture (Fig. 7A). A qualitative analysis of the ultrastructure was performed, detailing the most relevant parameters for AF and NP: lamellar organization, matrix density and collagen fibrils (Table 1, n = 3). In the control disc, namely in the AF area, a high organization of the collagen fibers was observed, and fibrils were orderly organized with a lamellar disposition. The collagen fibers were mostly homogenous. Also, a highly dense matrix was present in both AF and NP of the control disc. In the cavity disc, the lamellar organization of the AF fibers were partially lost, becoming more randomly distributed. There was also a significant loss of matrix in both the AF and NP area, and higher fiber heterogeneity in the cavity group. On the other hand, in discs treated with hMSCs (C + hMSCs), some lamellar organization was observed, as well as a more dense matrix in both AF and NP, more similar to the control group. In addition, the collagen fibers seem to be less heterogeneous, in both AF and NP, when compared to the cavity group. At the cellular level, both active and healthy cells and necrotic cells were found in all groups, although non-representative numbers of cells were observed. Healthy cells had signs of cell activity and matrix synthesis (normal cell nucleolus, intact cytoplasm and organelles; dense ECM matrix components encircling cells), while necrotic cells presented signals of chromatin clumping and, in some cases, cytoplasmatic deterioration (see Supplementary Data).

Collagen fibers in both AF and NP were further studied based on birefringence intensity using Picro-Sirius red staining (PSR). By this technique, the thick/mature fibers display an orange/reddish color under polarized light and the intermediate mature fibers display a yellow color (these two are often associated with Col type I), while the thin/immature fibers display a green color (generally associated with Col type III)^28^. Col type II, typically of cartilaginous tissues, such as the IVD, forms very small fibrils which are embedded in ground substance and therefore result in a weak birefringence of varying color^28^. A representative image of PSR staining can be observed in Fig. 7B. The different colors of the collagen fibers (red, yellow and green) were observed by polarized light and quantified accordingly (Fig. 7C). The green birefringence represented the smaller percentage of fibers, compared to yellow-reddish fibers in both AF and NP. The control disc, presented a balanced distribution of the three types of fibers. In the NP of the cavity and C + hMSCs groups, a higher percentage of mature fibers (red) was found, which might be explained by the expression of Col type I. By determining the ration of greenish/reddish fibers (Fig. 7D) we observed a decrease in both NP and AF of the cavity group (NP, 0.04 ± 0.02; AF, 0.07 ± 0.1), compared with control discs (NP, 0.5 ± 0.1; AF, 0.2 ± 0.02). This ratio slightly increased in the NP of the C + hMSCs group (NP, 0.09 ± 0.06; AF, 0.07 ± 0.03) suggesting higher synthesis of new fibers and thus corroborating the increase in Col type II in the NP of C + hMSCs group.

### Growth factor production in *ex vivo* IVD cultures with MSCs

**Figure 8:**
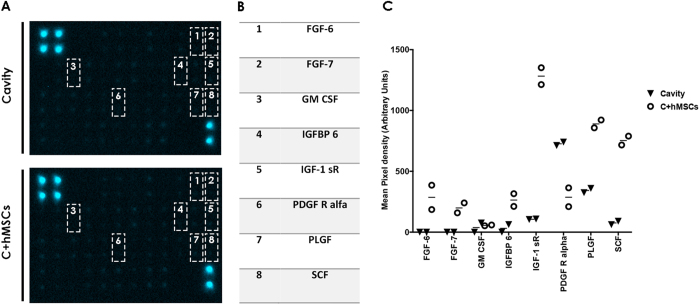
Growth factor production in the IVD culture. Growth factors production was evaluated using an array, in a pool of 6 donors. **(A)** Images of the array membranes obtained for each experimental group (cavity and C + hMSCs); **(B)** Table with the key detected cytokines. **(C)** Quantification of the key growth factors by quantifying the mean spot pixel density from the array. The densities of signals were normalized with the background. Results are presented as a plot of individual values (two dots). 7 key GFs were shown to be increased in the presence of hMSCs, while one of them decreased. In general, all the key GFs were directly or indirectly related with cartilage remodeling and ECM synthesis.

A screening of growth factors production was performed, in an attempt to unveil the mechanisms underlying NP matrix remodeling by hMSCs seeded in CEP. Organ culture supernatants of nucleotomized IVDs (cavity) and IVDs with hMSCs on CEP (C + hMSCs) at 21 days of culture were pooled (n = 6) and analyzed by an antibody array of 42 growth factors (see Supplementary Data). Seven growth factors were observed to be up-regulated in the medium of the C + hMSCs group while only one was up-regulated in the cavity group (Fig. 8A,B). However, it was not possible to distinguish between bovine or human proteins. The presence of hMSCs (C + hMSCs) considerably increased the levels of: i) fibroblast growth factors-family, FGF-6 and FGF-7, insulin-like growth factor binding protein 6 (IGFBP6) and Stem cell factor (SCF) (>6); ii) insulin-like growth factor 1 receptor (IGF-1 sR) (>4); iii) placental growth factor (PLGF) and granulocyte macrophage colony-stimulating factor (GM CSF) (>2). In addition, the presence of hMSCs decreased the levels of platelet-derived growth factor receptor, alpha (PDGF R alpha) compared to the cavity group (<0.5) (Fig. 8C).

## Discussion

MSCs are one of the most attractive cell types for IVD regeneration, as recently reviewed by Vadalà *et al*.^29^. Among the different sources of adult stem cells, including adipose, muscle or even IVD-derived stem cells, bone marrow (BM) derived MSCs are by far the most used source of cells. BM-MSCs have shown great differentiation capacity towards NP cells phenotype within multiple studies and have already been used in clinical trials, although the access to BM may entail risks and side effects. Nevertheless, adipose- and muscle-derived stem cells efficacy has not been established yet and IVD-derived stem cells are poorly characterized, lacking standardization of isolation methods and involve several risks in the harvesting procedure^29^.

The injection of hydrogel-encapsulated MSCs in the cavity of nucleotomized discs revealed that MSCs remain viable and are able to differentiate towards a disc-like phenotype^30^. Still, MSC administration via CEP aims to overcome the difficulties often associated with hydrogel injection in the NP (extrusion and cell leakage) in cell transplantation approaches, which are commonly performed via the AF and might lead to its rupture^16^,^17^. MSCs are able to migrate to injured tissues and act either on cell “replacement” (multilineage differentiation capacity) or on cell “empowerment” (release of immunomodulatory factors and/or growth factors)^31^. MSC migration towards the IVD was first reported by Junger *et al*.^26^ using an *ex vivo* model cultured under stimulated-degenerative conditions. Recently, using an *ex vivo* model of nucleotomized disc, we showed that hMSCs seeded on CEP could migrate towards the NP, and are additionally enhanced by SDF-1 incorporation into a hydrogel^27^. The advances in surgical techniques to access the NP avoiding AF damage, opened new perspectives for cell transplantation. Vadalá *et al*.^19^,^20^ reported a surgical transpedicular approach via the CEP as an alternative route for IVD regenerative strategies, in a sheep model.

The present study focused on the particular effect of hMSCs seeded on CEP in the IVD ECM remodeling. For that, an *ex vivo* model of nucleotomized IVD with CEP was used, which allows the study of IVD degeneration and regenerative strategies in a controlled manner, maintaining an intact AF^30^, while preserving the IVD morphology with the CEP. This model was previously used and maintained in culture for 2 days by our group^27^ and up to 7 and 14 days in culture by others^30^,^32^. In this study, long-term culture periods were fundamental to understand repair/regenerative processes at the protein level. Nonetheless, IVD long-term culture is a challenge due to the balance between nutrient supply and tissue structure maintenance, i.e. in prevention of swelling. The presence of the CEP in IVD cultures was shown to maintain IVD structure and to favor tissue viability, metabolism and to impair swelling and matrix degradation^33^,^35^. In this study, our model was maintained for 21 days. After this period, metabolic activity, cell proliferation and DNA content was firstly assessed. Globally, all the experimental groups were metabolically active, with low numbers of proliferative cells (<15%). Low cell proliferation rates appear to be common in both healthy and herniated human discs^36^. The Ki67^+^ proliferative cells were more frequently found in the cavity and C + hMSCs groups, suggesting that the injury by itself might stimulate cell proliferation and could represent a matrix repair response to the stimuli triggered by the cavity. This response, triggered by a stimuli, was reported by Johnson *et al*.^37^, in pathological human discs. The DNA levels were slightly higher in the hMSC treated group, suggesting higher cell content possibly due to cell migration from the CEP.

hMSC migration in the IVD tissue was confirmed after 21 days of culture, by detection of CM-Dil-labeled hMSCs (red) in both, AF and NP regions, namely in the lesion (cavity) area. Cell migration in the ECM occurs via degradation by MMPs or other proteolytic enzymes. Both MMP2 and MMP9, among others, have been associated with hMSCs invasion capacity^38^, while an increase in MMPs has been associated with IVD degeneration^39^,^40^. Herein, both MMP2 and MMP9 activities were evaluated. Although MMP2 and not MMP9 activity was detected, we could not distinguish between MMP production by hMSCs or bovine IVD cells. Hence, the mechanism by which cells migrate from the CEP to the IVD tissue is not fully elucidated. Future studies need to be designed to evaluate the contribution of MMPs and other enzymes in the migration process, in this particular model.

The effect of hMSCs seeded on CEP on the matrix of both NP and AF was then evaluated after 21 days in culture. hMSCs treatment through the CEP had a positive effect on proteoglycans, as suggested by the intense signal in the SO-FG staining in the IVDs of C + hMSCs treated discs, and an increased Agg expression in the NP, at both cellular and matrix level. Agg, the major proteoglycan constituent, has a key role in the maintenance of the osmotic properties of the IVD^41^. Agg cellular expression was quantified using ImmunoCellCount software, which allowed a faster and more accurate analysis of cell associated Agg deposition in the IVD tissue, thus encouraging the use of this software in future analysis to count Agg+ cells in IHC sections.

Previous studies have reported an upregulation of both Agg and Col type II gene expression in the presence of hMSCs, in *ex vivo*^30^ and *in vivo* setups^42^, for example in a porcine model^43^. Col type II is known to be present in both AF and NP, being more abundant in the NP area, while Col type I is the main component of the AF^1^. In this study, Col type II was also quantified and a significant in the NP of hMSCs-treated IVDs was observed, suggesting that the hMSCs were not only able to re-activate the synthesis pathways of Agg but also of Col type II. In addition, Col type I expression in the IVD tissue was analyzed. Col type I expression was more pronounced in the AF areas, decreasing towards the NP. Still, a higher expression of Col type I was observed in the NP of both lesion groups (cavity and C + hMSCs). In fact, the progression of the IVD degenerative process has been associated with alterations in collagen types distribution, such as an increase in Col type I in the NP area^44^, which is in accordance with the results observed in this model.

The ECM ultrastructure of bovine IVDs in culture was also explored. Dense ECM, namely collagenous fibrils and proteoglycans, and sparse AF/NP cells were observed, similarly to what was described for human IVD^45^. Higher ECM density was observed in both control and C + hMSCs groups with a more consistent fiber diameter being observed in control discs; while more heterogeneous fibers were observed in both cavity and C + hMSCs groups. Fiber heterogeneity present in the cavity and C + hMSCs groups might be due to tissue remodeling post-lesion. Smaller collagen fibrils suggests for the synthesis of new fibers and less uniform collagen fibers were previously described in human degenerated NP^46^. To further characterize these fibers, analysis and quantification of birefringent collagen fibers by the picrosirius-polarization method was performed^47^,^48^. This staining allows to distinguishes between mature and immature collagen fibers, although some authors also relate fiber polarization colours with collagen types^49^. Col type I is associated to the red/orange colours while Col type II presents variable colours depending on the tissue and species^50^. Higher percentage of red fibers was found in the NP of lesion groups (cavity and C + hMSCs), suggesting an increase in Col type I, corroborating our IHC findings. The highest ratio of greenish/reddish fibers was observed in the NP of control discs and a slightly higher amount of green fibers was observed in the hMSCs-treated group, comparing to the cavity, indicating an increase in the amount of immature fibers and thus suggesting *de novo* ECM synthesis. Still we cannot exclude the contribution of Col type II, significantly increased in the NP of C + hMSCs group, as this collagen type might also assume these polarization colors.

Behind the beneficial effect of MSCs on ECM of IVD could be their capacity of differentiation into IVD-like cells^43^. MSC differentiation towards NP-like cells has been described and associated with the production of insulin-like growth factor (IGF-1), basic fibroblast growth factor (FGF-2), platelet derived growth factor (PDGF)^51^, and growth and differentiation factor-5 (GDF 5)^52^. Herein, we have investigated the associated growth factors in IVD culture media using an antibody array, post treatment of nucleotomized discs with hMSCs. This analysis revealed that 7 (out of 42) growth factors were more concentrated in C + hMSCs group, while only 1 was increased in the cavity group. The majority of the growth factors detected are known to be involved in ECM synthesis. For example, FGF-6 was shown to support MSC chondrogenesis, together with transforming growth factor-beta-2 (TGF-beta2)^53^ and FGF-7 was suggested to induce chondrocyte proliferation^54^. A decrease in PDGFR alpha was detected in the C + hMSCs group: PDGF was shown to stimulate proteoglycan synthesis and cell proliferation in cartilage^55^ and PDGFR is down-regulated or lost in cartilage-forming areas, as chondrogenic differentiation occurs^56^, which could explain the results observed. In addition, IGF-1 receptor (IGF-1 sR), a key regulator of chondrogenesis and proteoglycan metabolism^57^, was also increased in the C + hMSCs group. IGFBP 6 is a factor involved in IGF transport in articular cartilage, highly abundant in bovine cartilage^58^,^59^; GM-CSF is a factor involved in Col type II and proteoglycan synthesis by rat chondrocytes^60^ and PLGF is commonly associated with bone remodeling/regeneration and cartilage turnover^61^. Importantly, we exclude hMSC differentiation towards osteogenic lineage by Alizarin staining (see supplementary data). SCF, which was also augmented in the C + hMSCs group, has been described as a key stimulus in the regulation of proteins involved in hMSC proliferation and chondrogenesis^62^. Overall, all the growth factors identified correlate well with an augmented production of cartilaginous ECM. Still, we cannot exclude whether other growth factors might be highly expressed inside the IVD tissue, or if these growth factors were produced by hMSCs or by IVD cells stimulated by hMSCs. Nevertheless, our data suggests that hMSCs in CEP contribute to IVD ECM remodeling via a paracrine effect and that the beneficial presence of hMSCs relies on a combination of different growth factors, cytokines and other molecules. In the future more studies should be conducted to dissect which IVD cell subsets are specific targets of MSCs.

Although a clear increase of Agg and Col type II expression/synthesis in the NP was observed with our approach, we cannot exclude that ECM remodeling could be further enhanced by injection of growth factors such as bone morphogenic proteins (BMPs), transforming growth factor-β (TGF-β), growth/differentiation factor 5 (GDF-5) which could stimulate ECM production and cell proliferation^63^, or even using short peptides such as LinkN, which have shown similar effects to MSCs injection in IVD ECM^64^. The use of techniques such as the “priming” of MSCs by cell transfection with non-viral vectors with GDF-5^65^, or mechanical stimulus in bioreactors, which could provide specific mechanical or hydrostatic loading and hypoxia, could also benefit our strategy^66^. Besides, we cannot exclude that our results could be further enhanced by maintenance of IVD cultures under dynamic loading.

The CEP structure has a key role in IVD homeostasis by balancing the nutrition through its porous structure and by providing stiffness to resist axial loading^67^. Although it can represent an alternative route to degenerated IVD treatment by avoiding the injection through AF, this approach might be limited. For example, in patients where degeneration was initiated due to the blockage of nutrients diffusion in the CEPs, as in some trauma patients^66^ or in patients with double-layer CEPs with increasing thickness^68^, where the capacity of cells to migrate might be impaired. In those cases, other surgical approaches need to be developed to unblock the CEPs, such as the microfracture approach currently applied to stimulate articular regeneration^69^. To date, it remains unclear at which degeneration level should MSCs-based therapies be applied, but we believe that success would be increased if applied in early degenerative levels in an attempt to repair or delay further degeneration.

This work supports the concept of MSC migration to promote regeneration of degenerated IVD, as an alternative to cell transplantation, using the CEP approach as a main route for MSCs.

## Conclusions

This work provides new insights on cell therapies and ECM remodeling in the IVD. The results obtained suggest that stem cell migration to IVD can improve local cell activity and ultimately tissue repair through the synthesis of matrix components. This reinforces the relevance of alternative strategies to MSC transplantation, such as the injection of molecules, which can trigger endogenous cell migration from local niches. Future studies will improve our understanding of the signaling pathways behind hMSCs effect in the IVD and reveal the relevance of MSC recruitment to IVD by chemoattractors for the spine field.

## Materials and Methods

### Human Mesenchymal Stem Cells Culture

hMSCs were obtained from discarded human BM tissues from patients undergoing total hip arthroplasty. Patients gave informed written consent for tissue use for research purposes and procedures were carried out in accordance with the relevant guidelines approved by the Centro Hospitalar São João Ethics Committee. All samples were analyzed with patient data coded. hMSCs were obtained from two different donors with ages of 45 and 49 years and isolated by density gradient centrifugation and adherence to tissue culture plastic as previously described^70^. MSC isolation was confirmed by flow cytometry (cells were CD105, CD73 and CD90 positive, while CD45, CD34, CD14, CD19 and HLA-DR negative), and capacity to differentiate into osteoblasts, chondroblasts or adipocytes^70^ (data not shown). hMSCs were expanded in low-glucose Dulbecco’s modified Eagle medium (DMEM, Gibco) containing 10% FBS and 1% Penicillin/Streptomycin (P/S) until reaching the total number of cells needed (1 × 10^6^ cells/disc), in general in passages P4 to P8.

### Intervertebral disc isolation

Bovine IVDs were isolated from young adult animals’ tails (5–10 months old) within 3 hours post-slaughter in a local slaughterhouse (Carnes Landeiro, Barcelos, Portugal). All experiments were performed in accordance with relevant guidelines and regulations, with the ethical approval of the Portuguese National Authority for Animal Health. IVDs with CEPs were harvested in sterile conditions following a protocol previously described^27^. Briefly, the caudal discs with CEPs were removed using a band saw (Dremel ^®^ Moto-Saw (MS20-1/5)) to obtain parallel cuts. The CEPs were afterward jet-lavaged with sterile phosphate-buffered saline solution (PBS, pH 7.4), using a Pulsavac wound debridement irrigation system (Zimmer, Inc., Switzerland). Discs were washed sequentially in 1%/10%/1% of P/S in PBS (pH 7.4) for 1/10/1 min, respectively. Afterwards, discs were incubated overnight in 6-well plates with high-glucose Dulbecco’s modified Eagle medium (DMEM, Gibco) supplemented with 2% fetal bovine serum (FBS, Gibco), 1% P/S (Gibco), 1% insulin transferrin selenium supplement (ITS) (BD, Becton Dickinson) and 0.1% Primocin (Invivogen) at 37 °C in a 5% CO_2_ atmosphere.

### Intervertebral disc Culture

**Figure 9:**
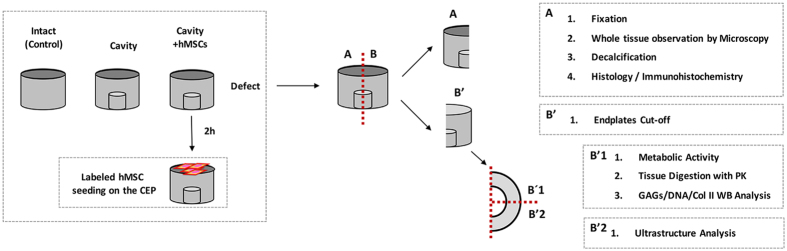
Schematic representation of the experimental setup.

A previously described model of IVD nucleotomy accessed through the CEP was adopted to mimic the loss of ECM by the removal of part of the NP^27^,^30^. Briefly, a circular cavity was made in the CEP. After a portion of the CEP was removed, part of the NP (0.05–0.1 cm^3^ of tissue) was removed using a blade. The removed CEP was afterwards repositioned, sealed with bone cement (PMMA, Vertecem V Cement Kit, Synthes, Switzerland). Finally, all the discs were turned to place the cavity at the bottom of the well and incubated in medium (DMEM 4.5 g/L glucose, 2% FBS, 1% P/S, 1% ITS and 0.1% Primocin) in 6-well plates for 2 h before cell seeding. In these setup, two experimental groups were defined, the “cavity” disc with an empty cavity and the “C + hMSCs” disc, a cavity disc with seeded hMSCs on the CEP (Fig. 9). Intact discs (discs without cavity) were used as a control group.

hMSCs were seeded in the C + hMSCs group, at a density of 1 × 10^6^ cells/disc^27^, on top of the CEP (i.e. on the side opposite to the injury) and incubated for 30 min to allow cells to enter the CEP structure. Afterwards, medium (DMEM 4.5 g/L glucose, 2% FBS, 1% P/S, 1% ITS, 0.1% Primocin) was added and discs were cultured at 37 °C, in 5% CO_2_ atmosphere, during 21 days. Media were exchanged every 2 days. After this period, discs were extensively washed with PBS and cut transversally with a blade, or sagittaly (with CEP) using a Dremel^®^ Moto-Saw (MS20-1/5). Discs were then stored at −20 °C or fixed in 4% buffered formalin for 3–4 days, for further analysis. Independent experiments were performed using hMSCs from two different donors (n = 2) and ten animals (n = 10). An additional condition (n = 3) using labeled hMSCs was used for cell tracking after 21 days in the tissue. hMSCs were previously labeled with CellTracker™ CM-DiI Dye (Thermo Fisher Scientific Inc.) following the manufactures’ instructions. The whole IVD sagittal section (Fig. 9A) was imaged by high-content analysis system using In Cell Analyzer 2000 (GE Healthcare Life Sciences). Discs with non-labeled hMSCs were used as control.

### Intervertebral disc Culture: metabolic activity, viability and cell proliferation analysis

Cellular metabolic activity of the disc tissue was measured using resazurin sodium salt assay as previously described by others^71^,^72^. Briefly, at day 21, discs were cut in half (Fig. 9B) and the CEP removed in one of the half (Fig. 9B′), remaining the AF and NP. This half was further divided in ¼ (Fig. 9B′1) and incubated in a 6-well plate, with 10 mL of 10% resazurin solution (0.1 mg/mL resazurin sodium salt (C_12_H_6_NNaO_4_, Sigma) in PBS) in DMEM 4.5 g/L glucose, 2% FBS, 1% P/S, 1% ITS, 0.1% Primocin during 3 h, at 37 °C in a 5% CO_2_ atmosphere. Relative Fluorescence unit (RFU) was measured at an excitation wavelength of 530 nm and an emission wavelength of 590 nm using a microplate reader, Synergy™ Mx multi-mode microplate reader (BioTek^®^ Instruments, Inc., Vermont, CA). Cell metabolic activity was expressed as RFU normalized to DNA. A blank control comprising only medium was also included.

Cell proliferation was detected by IF, using Ki67 proliferation marker. Masked epitopes were exposed by treatment with 10 mM sodium citrate (pH 6) for 35 min at 95–98 °C. Paraffin sections were incubated overnight (4 °C) with rabbit anti-Ki67 primary antibody (ab15580, Abcam plc, 330 Science Park, Cambridge CB4 0FL, United Kingdom, 1:100). Alexa Fluor 488-labeled goat anti-rabbit was used as a secondary antibody. All sections were mounted in Fluorshield with DAPI (Sigma). Control sections for each immunolabeling excluded primary antibody staining. Representative images were taken in an inverted fluorescent microscope (Axiovert 200M, Zeiss) with 40x and 100x objectives. The number of proliferative cells (Ki67+) was assessed for each group and counted manually on ImageJ 1.43u software (Wayne Rasband, National Institutes of Health, USA).

### DNA Quantification

DNA content in the IVD tissue was quantified using a CyQuant^®^ kit (Invitrogen). Briefly, 100 mg of NP tissue previously frozen at day 21 were minced into very small pieces and then digested in a proteinase K solution (0.5 mg/mL) overnight at 56 °C. DNA content was expressed in μg according to standard and normalized to the wet weight (mg) of the digested tissue.

### Histology

IVDs with CEPs were harvested after 21 days of culture and fixed in 4% buffered-formalin. For haematoxilin & eosin (H&E) and safranin O-Fast green (SO-FG) staining, discs were cut sagittaly with CEPs and decalcified with 17% neutral ethylenedinitrilo-tetraacetic acid trisodium salt (EDTA, pH 7.0) for 10 days (EDTA solution was changed twice), and afterwards embedded in paraffin. Paraffin sections were cut in the sagittal plane at 3 μm. H&E was performed for an overall assessment of the histological structure. Sections were incubated in Gill’s haematoxilin (Sigma-Aldrich) for 5 min to stain the cell nuclei, washed and dehydrated through graded alcohol solutions previous to a counterstain in alcoholic eosin (Surgipath) for 1 min, to detect cell cytoplasm and most connective tissue fibers. SO-FG was performed for assessment of matrix deposition in the tissue. Briefly, sections were incubated in Gill’s haematoxylin (Sigma-Aldrich) for 5 min to stain the cell nuclei and, afterwards, immersed in 0.4% FG (Sigma) solution during 5 min to stain the collagen. After washing twice in 1% acetic acid solution, slides were immersed for 4 min in 0.1% SO (Sigma-Aldrich) solution to detect proteoglycans deposition. Sections were imaged with an Olympus CX31 light microscope equipped with a DP-25 camera (Imaging Software CellˆB, Olympus, Center Valley, PA, USA) at different magnifications (10x, 40x and 100x).

### Sulphated Glycosaminglycans quantification

GAG content in the IVD tissue was assessed in Proteinase K digested IVD tissue, as previously described for DNA quantification. GAG were quantified by the reaction with 1,9-Dimethyl-Methylene Blue zinc chloride double salt (DMMB, Sigma-Aldrich) dye reagent solution, containing 40 mM sodium chloride (NaCl, Roth), 40 mM Glycine (Roth) and 46 μM DMMB, previously adjusted to pH 3.0 as described^71^. Chondroitin sulphate A sodium salt from bovine trachea (Sigma) was used as standard. GAG content was expressed as μg/mg (wet weight) according to the standard. Results were normalized and presented as a fold increase to the cavity disc (n = 4).

### Collagen type II quantification

Col type II quantification in the IVD tissue was assessed by western blot (WB). Briefly, part of previously frozen NP was minced and incubated with an optimized buffer for protein extraction containing 4 M guanidine hydrochloride (Sigma), 3 M sodium acetate (Merck) and 10 mM EDTA, and enriched with a protease and phosphatase inhibitor cocktail (Roche Diagnostics GmbH, Manheim, Germany and Sigma, respectively). Protein quantification was performed using the 2-D Quant Kit (GE Healthcare) according to the manufacturer’s instructions. Protein samples (20 μg) were afterwards separated by sodium dodecyl sulphate (SDS)/9% polyacrylamide gel electrophoresis, and electroblotted onto a Hybond enhanced chemiluminescence (ECL) membrane (Amersham BioscienceseGE Healthcare). The monoclonal antibody against Col type II (1:1000 dilution) (II-II6B3) was used with a sheep anti-mouse (1:3000 dilution; Amersham Biosciences) horseradish peroxidase-conjugated secondary antibody, followed by ECL detection (Amersham Biosciences). Bands were quantified using Quantity One^®^ 4.6.6 Software (Bio-Rad, Amadora, Portugal). Values were normalized to the density of each corresponding complete lane (total protein loaded)^73^. Results were afterwards normalized to the cavity group and presented as a fold increase (n = 7).

### Immunohistochemistry

For IHC and IF techniques, non-decalcified IVD paraffin sections without CEPs were used. Agg and Col type I expression in the different IVD regions (NP and AF) was assessed by IHC. NovolinkTM Polymer Detection Kit (Leica Biosystems, Newcastle, UK) was used, following the manufacturer’s instructions. Antigen retrieval was performed through the incubation with a 20 μg/mL proteinase K (Sigma-Aldrich) solution for 15 min at 37 °C. For Col type I sections were boiled in citrate buffer (pH 6.0), as pre-treatment and then incubated with 1.5 U/mL solution of hyaluronidase (Sigma-Aldrich). After neutralization of endogenous peroxidase using Peroxidase Block for 5 minutes, a blocking step was performed. Sections were afterwards incubated with the primary antibody Agg (H-300) sc-25674 (Santa Cruz Biotechnology, Inc, Texas, USA) (1:50) or Col type I primary antibody (1:100) 600-401-103-0.1 (Rockland Immunochemicals, Inc. Limerick, PA), overnight. Bound antibodies were revealed after a 30 min incubation with Novolink^TM^ Polymer in the dark and 5 min incubation with peroxidase-substrate solution DAB. A negative control was performed in each slide without the primary antibody. Representative images of the slides were taken using an Olympus CX31 light microscope (20x objective for counting and 100x oil objective for detailed imaging of Agg IHC; 10x for Col I IHC). Agg and Col type I matrix staining in the different regions of the IVD (NP and AF) was qualitatively assessed by two independent observers. Agg cellular staining was quantified using a custom-made software written in MATLAB (The MathWorks Inc., Natick MA, USA) named ImmunoCellCount (see Supplementary Data) (n = 5).

Col type II distribution was analyzed by IF staining. For IF, antigen retrieval was performed in paraffin sections through incubation with a 20 μg/mL proteinase K (Sigma-Aldrich) solution for 15 min at 37 °C. After a blocking step, sections were then incubated for 2 h at 37 °C with the primary antibody against Col II (1:50) (monoclonal antibody against Col type II (II-II6B3) developed by Dr. Thomas F. Linsenmayer, from the Developmental Studies Hybridoma Bank under the auspices of the NICHD and maintained at the University of Iowa, Department of Biology, Iowa City, IA 52242). Alexa Fluor 594-labeled goat anti-mouse (Invitrogen-Molecular Probes, 1:1000) was used as the secondary antibody for Col II detection during 1h, room temperature, in the dark. All sections were mounted in Fluorshield with DAPI (Sigma). Control sections for each immunolabeling excluded primary antibody staining. Representative images were taken using an inverted microscope, Axiovert 200 M, Zeiss. Col II intensity in the different regions of the IVD (NP and AF) was quantified using a custom-made software written in MATLAB (The MathWorks Inc., Natick MA, USA) named IntensityStatisticsMask (see Supplementary Data) (n = 5).

### Ultrastructure Analysis by Transmission electron microscopy (TEM)

Previously fixed portions of IVD tissue from each experiment were further processed for TEM. The different IVD regions, AF and NP were separated and cut into very small pieces. These samples were washed in PBS and fixed in 2.5% glutaraldehyde and 2% paraformaldehyde in 0.1 M sodium cacodylate (pH 7.4) for 2 h. After washing in 0.1 M sodium cacodylate buffer for 30 min the tissue was fixed in 2% (v/v) osmium tetroxide in 0.1 M sodium cacodylate overnight followed by another fixation with 1% uranyl acetate overnight. Samples were dehydrated in gradient series of ethanol solutions as follows: 50% ethanol for 10 min, followed by 70%, 80%, 90%, 96%, 100% and propylene oxide (v/v). Inclusion in EPON resin was performed by immersion of tissue in a gradually increasing series of propylene oxide to EPON as follows: 2:1, 1:1, 1:2 and 0:1 for 60 min each. At the end, inclusion of the tissue in EPON resin was performed in a silicon mold. EPON polymerization took place at 60 °C for 48 h. Sections with 50 nm thickness were prepared using a diamond knife (Diatome, Hatfield, PA, USA) and were recovered to 200 mesh Formvar Ni-grids. Staining of sections using 2 wt% uranyl acetate and saturated lead citrate solution, for 7 min each, was performed before observation. Visualization took place at 80 kV in a (JEOL JEM 1400 microscope (Japan)). Both AF and NP from the different bovine donors were analyzed per group (cavity and C + hMSCs), and a freshly isolated disc was used as a control. 20–30 images for each sample were analyzed by two independent observers (n = 3). A qualitative analysis of several parameters was performed based on previous studies^46^ and categorized by us in a scoring system (Table 1) in what concerns to: i) lamellar organization of the AF (highly organized (++++), disorganized (+)); ii) matrix density (higher matrix density (++++), sparse matrix (+)); iii) collagen fibers size heterogeneity (from high to low). The cellularity of the tissue, i.e. the presence of cells and their activity (very active, synthetic activity or necrotic) was also accessed (see Supplementary Data).

### Picrosirius-polarization method and quantification of birefringent fibers

The picrosirius-polarization method and consequent birenfrigent fibers quantification was performed in IVD tissue sections to assess the structural changes based on the birefrigence of the collagen fibers^48^. Sections of 3 μm were simultaneously stained with Sirius Red solution for 1h to avoid variations. Picrosirius Red stained sections were analyzed through a polarizing lens and all images were captured with the same parameters. A color threshold was applied using ImageJ software (version: 148r) in order to identify and quantify red, yellow and green fibers (n = 3).

### Growth factors Analysis in IVD culture media

A commercially available array of 42 growth factor proteins (RayBio^®^ C-Series Human Growth Factor Antibody Array C1, #AAH-GF-1-4, RayBiotech, Inc. 3607 Parkway Lane, Suite) was used to evaluate the relative levels of growth factors production in the IVD culture media of both cavity and C + hMSCs at day 21. A pool of culture media collected from 6 independent experiments was prepared for this assay, and 1 mL of the prepared pool was used. The array was performed by following the manufacturer’s instructions. Data shown represents 5 min exposure in Chemidoc XRSþ (BioRad). Results were generated by quantifying the mean spot pixel density from the array using image software analyses (ImageLab 4.1; BioRad). The densities of signals obtained were normalized with the background and results presented as a mean of two spots per growth factor.

### Statistical Analysis

Statistical analysis was conducted using GraphPad Prism version 6.0f for Mac OS X (GraphPad Software, California, USA). The non-parametric Mann-Whitney test was used to compare two groups of non-related samples. Statistical significance was considered whenever *p < 0.05; **p < 0.01; ***p > 0.001 and when p ≥ 0.05 as not significant (NS).

## Additional Information

**How to cite this article**: Pereira, C. L. *et al*. Mesenchymal Stem/Stromal Cells seeded on cartilaginous endplates promote Intervertebral Disc Regeneration through Extracellular Matrix Remodeling. *Sci. Rep.* **6**, 33836; doi: 10.1038/srep33836 (2016).

## Supplementary Material

Supplementary Information

## Figures and Tables

**Table 1:** Qualitative analysis of the main features of IVD ultrastructure.

Parameters	Lamellar Organization	Matrix Density		Collagen Fibers	
IVD Area	AF	AF	NP	AF	NP
Control	+++	+++	+++	Low Variation	Low Variation
Cavity	+	++	+	Moderate Variation	High Variation
C+hMSCs	++	+++	++	Moderate Variation	Moderate Variation
