# Supplementary material for: Mesenchymal Stem/Stromal Cells seeded on cartilaginous endplates promote Intervertebral Disc Regeneration through Extracellular Matrix Remodeling
Source: Sci Rep. 2016 Sep 22;6:33836. doi: 10.1038/srep33836 (PMC5031983; doi:10.1038/srep33836)
Supplement: Supplementary Information [file srep33836-s1.doc]

**Mesenchymal Stem/Stromal Cells seeded on cartilaginous endplates promote Intervertebral Disc Regeneration through Extracellular Matrix Remodeling**

**Catarina Leite Pereira^1,2,3^, Graciosa Q. Teixeira^1,2,3^, Cláudia Ribeiro-Machado^2,3^, Joana Caldeira^2,3,7^, Madalena Costa^6^, Francisco Figueiredo^2,5^, Rui Fernandes^2,5^, Paulo Aguiar^2,3^, Sibylle Grad^4^, Mário A. Barbosa^1,2,3+^ & Raquel M. Gonçalves^2,3+*^**

^1^ICBAS, Instituto de Ciências Biomédicas de Abel Salazar, Universidade do Porto, Porto, 4050-313, Portugal

^2^Instituto de Investigação e Inovação em Saúde, Universidade do Porto, Porto, 4200-135, Portugal

^3^INEB, Instituto de Engenharia Biomédica, Universidade do Porto, Porto, 4200-135, Portugal

^4^AO Research Institute Davos, Davos, 7270, Switzerland

^5^IBMC - Instituto de Biologia Molecular e Celular, Porto 4150-180, Portugal

^6^UMIB – Unit for Multidisciplinary Biomedical Research of ICBAS, Universidade do Porto, Porto, 4050-313, Portugal

^7^IPATIMUP – Institute of molecular pathology and immunology of the university of Porto, Porto 4200-135, Portugal

**^*^raquelg@ineb.up.pt**

**^+^This authors contributed equally to this work**

**Supplementary Data**

**1. hMSCs viability and survival in IVD culture media containing 2% of hMSCs tested serum.** hMSCs were cultured in the normal medium for hMSC expansion (low-glucose Dulbecco's modified Eagle medium (DMEM, Gibco) and 1% Pen/Strep) containing 10% of MSC tested FBS. As a control for cell survival and viability, a preliminary experiment was performed using the IVD medium (DMEM 4.5 g/L glucose, 1% Pen/Strep, 1% ITS, 0.1% Primocin) which contains only 2% of FBS. hMSCs were cultured during 48h in these two media and afterwards, cell death and apoptosis were assessed by FACS using Ethidium and Annexin V FITC staining. No differences were observed in cell death and apoptosis levels when cultured with 10% FBS or 2%FBS (Fig. 1S), giving indications that a low % of FBS in IVD culture would not compromise hMSCs viability in the IVD organ culture.

**
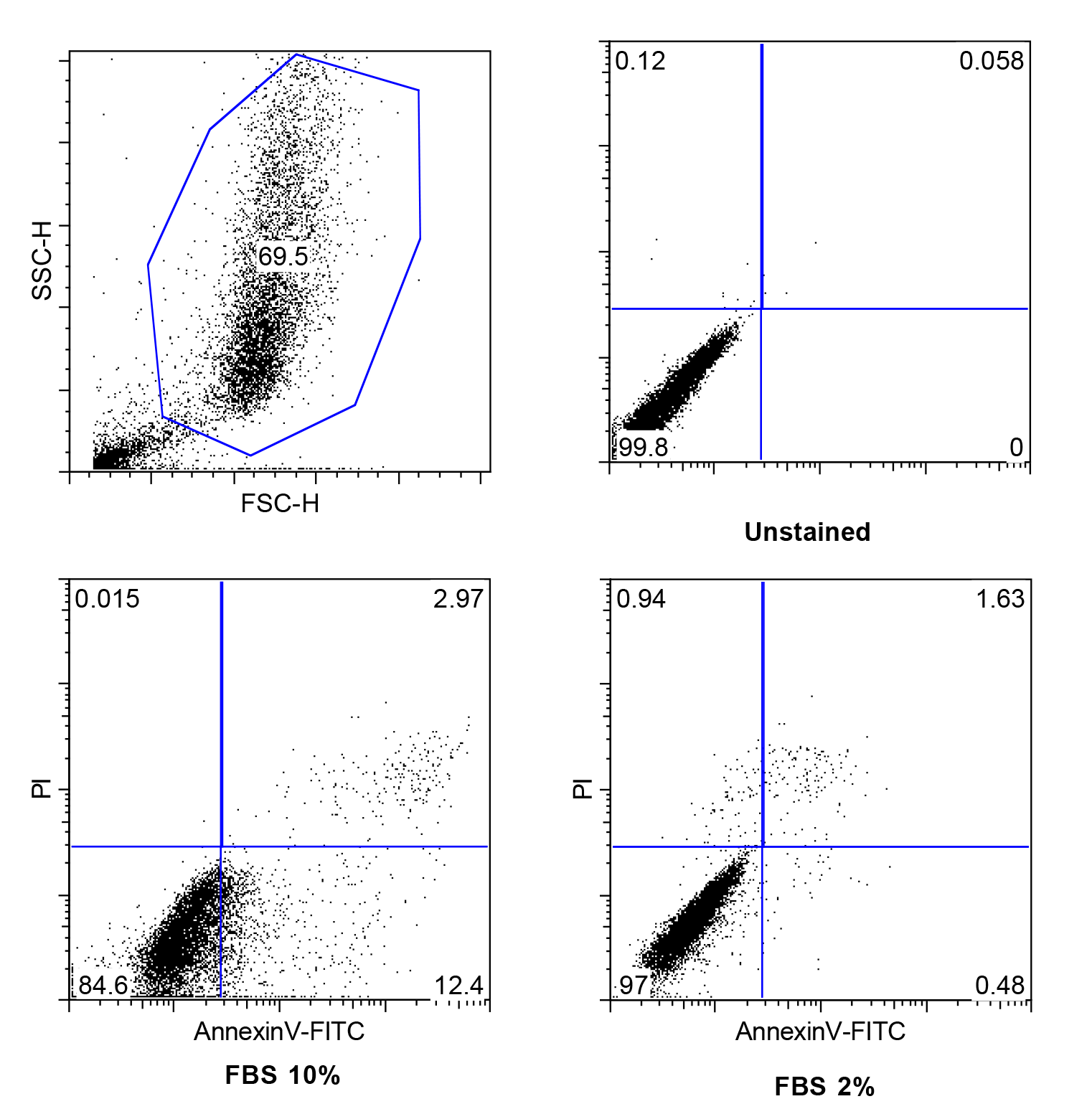
**

**Fig 1S – hMSCs apoptosis analysis in MSC and IVD culture media.**

**2. Metalloproteinases Quantification.** IVDs culture media was collected and analyzed by zymography at different time points. Matrix metalloproteinases (MMPs) activity, namely of MMP-2 and MMP-9 was assessed by gelatin zymography as previously described (Cardoso, A.P. et al. Macrophages stimulate gastric and colorectal cancer invasion through EGFR Y(1086), c-Src, Erk1/2 and Akt phosphorylation and smallGTPase activity. Oncogene, 2014). Levels of pro-MMP2 and MMP2 were augmented in C+hMSCs group. No pro-MMP and MMP9 were detected in the samples in the different time points.

**A**  **
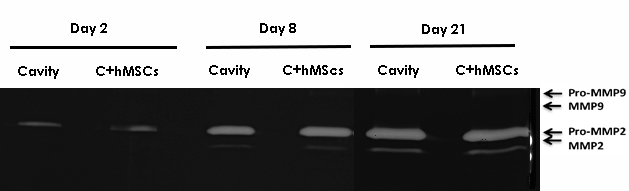
**

**B**
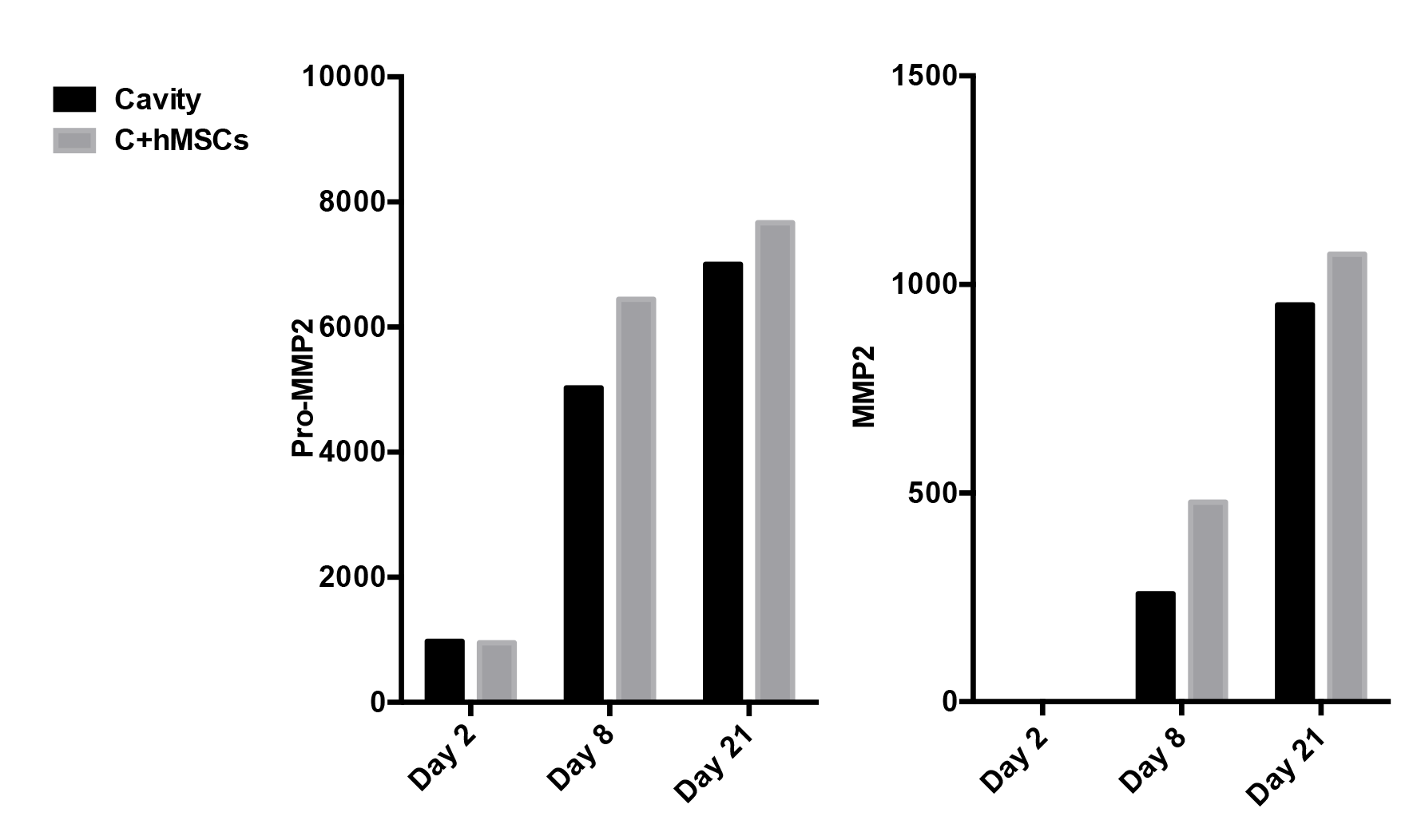


**Fig 2S - MMP2 and MMP9 activity in IVD culture media overtime**. **A:** Zymogram gel at different time points. The variants of MMPs are shown on the right. **B:** The intensity of gelatin digested bands of pro-MMP2, MMP2 were measured by densitometry and represented in the diagram bar.

**3. Calcification in IVD tissue.** The transplantation of MSCs into the IVD is sometimes associated with the presence of calcifications resulting from a differentiation towards a more osteogenic lineage over a disc-like/chondrogenic differentiation. To discard this hypothesis, we have performed a common staining for the identification of calcifications in the tissue, alizarin staining, on non-decalcified IVDs. Representative images of the staining can be observed in Fig. 3S. A dot-deposition of calcium was observed in the AF of 2/3 of animals analyzed, unrelatedly with presence of hMSCs. No calcifications could be found in the NP area in any group. Therefore, it is our believe, that those calcifications were inherent to the isolated discs from each animal, and not a result of a hMSCs differentiation.


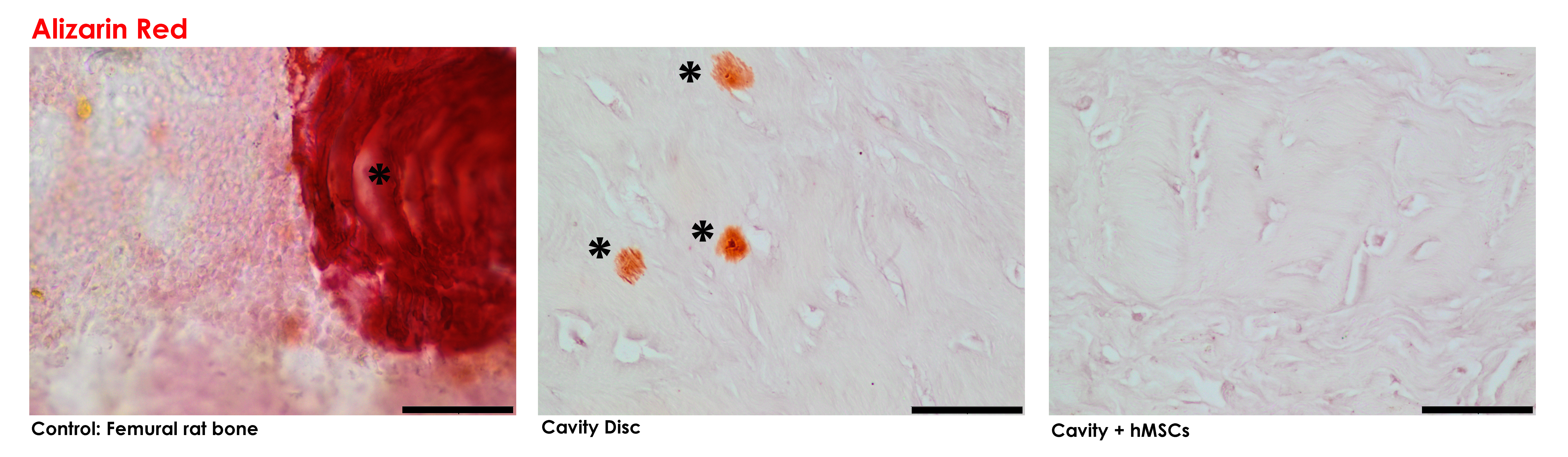


**Figure 3S - Alizarin staining in IVD tissue.** Alizarin (red*) staining in femural rat bone (positive control, left). Representative imagens of one animal in the AF area. Both in cavity discs (middle) and discs treated with hMSCs (right), calcification dots could be observed in the AF, but not in the NP. (Scale bar = 50 μm).

**4. Aggrecan IHC quantification**. IHC was performed in IVD section and aggrecan deposition was found to be co-localized with cells or in the very near neighborhood. Images were captured with an Olympus CX31 light microscope equipped with a DP-25 camera (Imaging Software CellˆB, Olympus, Center Valley, PA, USA) with the 20x objective to have a compromise between a statistically relevant number of cells and a proper color representation of the cells (provided by a sufficient number of pixels associated with every cell). IHC positive cells (Agg+) assume a brownish color while negative cells are purplish, allowing distinction between the two classes. The total number of cells and the number of Agg+ cells were determined either manually or using the ImmunoCellCount Software (Figure 2S) in a set of images.

**4.1. Software design.** The ImmunoCellCount software was implemented in MATLAB with the objective of developing an approach for unbiased, consistent, automatic and faster counting of Agg+ cells in IHC images of IVDs. This software provides a simple and user-friendly graphical user interface (GUI), and works in two stages: the first is responsible for cell identification (segmentation), and the second is responsible for cell classification. Two parameters are required for cell identification: an intensity threshold (for the segmentation algorithm) and a cell size (for object enhancement through a Laplacian of Gaussian filter). Cell classification is performed by measuring distances in color space: the user sets the reference colors for a negative cell (purple) and a positive Agg+ cell (brown); all cells found in the first stage are classified according to which reference color they are closer to (measured in RGB color space). All parameters, including reference colors, only need to be set once for a set of images sharing the same acquisition conditions. This method provides an unbiased and consistent procedure to classify and count positive and negative staining. To validate ImmunoCellCount, 12 images (more than 500 cells) were analyzed manually (detailed visual inspection of color) and with the software.

**5. Collagen type II quantification.** IF was performed in IVD section and collagen expression intensity was quantified in the images in both AF and NP areas of the disc. Images were captured using an inverted microscope, Axiovert 200 M, Zeiss with the 10x objective. The intensity of collagen expression in the tissue was determined using an additional custom made MATLAB script, the IntensityStatisticsMask Software.

**5.1 Software design.** The purpose behind the construction of this script was also to obtain a faster and unbiased method, now for color intensity quantification. The script performs image segmentation, based on a user-defined threshold level, to create a mask for the tissue regions expressing collagen. Intensity measurements, such as mean values and standard deviation, are then calculated only for the pixels belonging to the collagen mask. Unless otherwise stated, the intensity threshold value used for the segmentation was 10.

**6. Transmission Electron Microscopy.** The ultrastructure characterization of the IVD by transmission electron microscopy (TEM) allows a more profound visualization of matrix/cell alterations that occur during the degenerative process. At cellular level, both active and healthy cells and necrotic cells could be observed in all the groups, although non-representative numbers of cells were observed. Healthy cells had signs of cell activity and matrix synthesis (normal cell nucleolus, intact cytoplasm and organelles; dense ECM matrix components encircling cells) (Fig 4S A), while necrotic cells presented signals of chromatin clumping and, in some cases, cytoplasmatic deterioration (Fig 4S B).

**
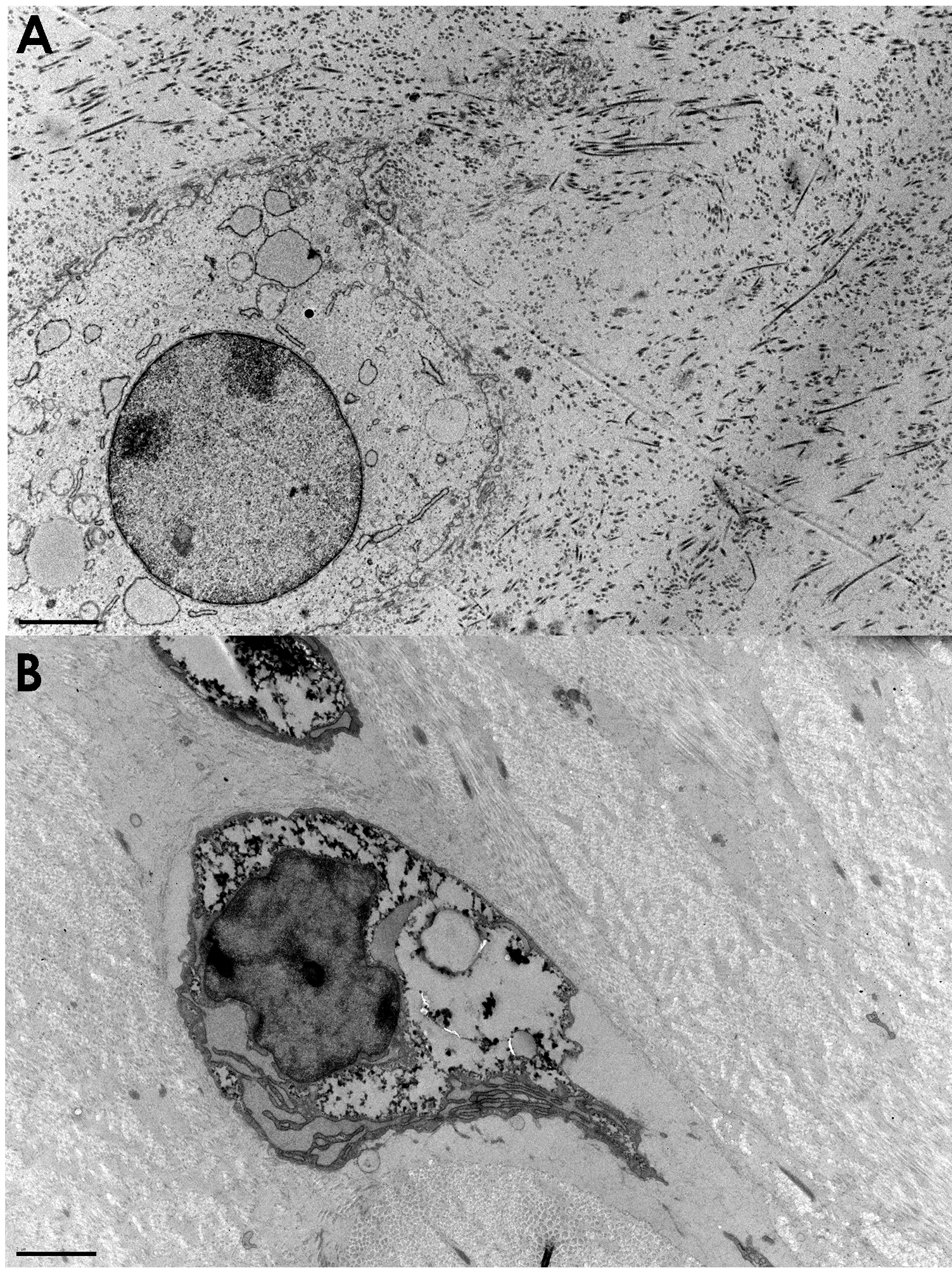
**

**Fig 4S – IVD cells observed by TEM.** A: healthy cell. B: necrotic cell**.**

**7. Growth Factors Analysis.** A commercially available array of growth factor proteins (RayBio® C-Series Human Growth Factor Antibody Array C1, #AAH-GF-1-4, RayBiotech, Inc. 3607 Parkway Lane, Suite) was used to evaluate the relative levels of growth factors production in the IVD culture media of both Cavity and C+hMSCs at day 21. The array is composed of 42 GFs, as depicted in Fig. 5S. A pool of 6 donors from the Cavity and C+hMSCs was analyzed. Results were generated by quantifying the mean spot pixel density from the array using image software analyses (ImageLab 4.1; BioRad). Briefly, the pixel intensities gathered from the array spots were obtained using the volume tools option of the software. We have defined an area of interest of the reference spots by surrounding it with a circle, and then equal circles were used for all spots of the array. The densities of signals obtained were normalized with the background. Results were afterwards represented as a color gradient of ranges of growth factors levels released in the IVD culture media. Each color representing a range of concentrations as indicated on the right table. For the results interpretation we considered alterations of a fold change >2 or <0.5.

**
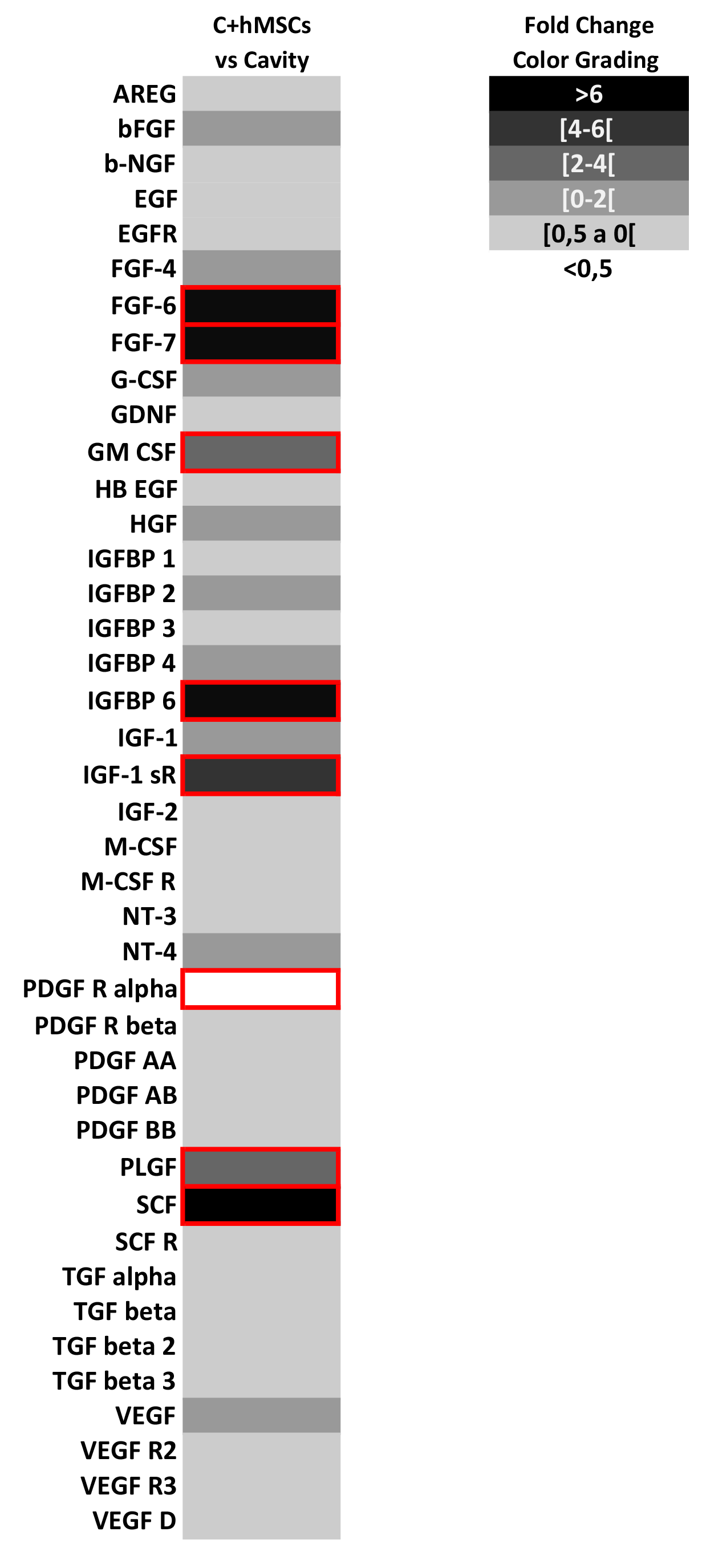
**

**Fig. 5S - Color gradient representation of ranges**
